# Knowledge and Awareness about Cervical Cancer and Its Prevention amongst Interns and Nursing Staff in Tertiary Care Hospitals in Karachi, Pakistan

**DOI:** 10.1371/journal.pone.0011059

**Published:** 2010-06-10

**Authors:** Syed Faizan Ali, Samia Ayub, Nauman Fazal Manzoor, Sidra Azim, Muneeza Afif, Nida Akhtar, Wassi Ali Jafery, Imran Tahir, Syed Farid-ul-Hasnian, Najam Uddin

**Affiliations:** 1 The Aga Khan University, Karachi, Pakistan; 2 Department of Community Health Sciences, The Aga Khan University, Karachi, Pakistan; Health Canada, Canada

## Abstract

**Background and Objective:**

Cervical cancer is one of the leading causes of morbidity and mortality amongst the gynecological cancers worldwide, especially in developing countries. It is imperative for at least health professionals in developing countries like Pakistan to have a sound knowledge about the disease. This study was carried out to assess the knowledge and awareness about cervical cancer and its prevention amongst health professionals in tertiary care hospitals in Karachi, Pakistan.

**Methods and Design:**

A cross-sectional, interview based survey was conducted in June, 2009. Sample of 400 was divided between the three tertiary care centers. Convenience sampling was applied as no definitive data was available regarding the number of registered interns and nurses at each center.

**Results:**

Of all the interviews conducted, 1.8% did not know cervical cancer as a disease. Only 23.3% of the respondents were aware that cervical cancer is the most common cause of gynecological cancers and 26% knew it is second in rank in mortality. Seventy-eight percent were aware that infection is the most common cause of cervical cancer, of these 62% said that virus is the cause and 61% of the respondents knew that the virus is Human Papilloma Virus (HPV). Majority recognized that it is sexually transmitted but only a minority (41%) knew that it can be detected by PCR. Only 26% of the study population was aware of one or more risk factors. Thirty seven percent recognized Pap smear as a screening test. In total only 37 out of 400 respondents were aware of the HPV vaccine.

**Conclusion:**

This study serves to highlight that the majority of working health professionals are not adequately equipped with knowledge concerning cervical cancer. Continuing Medical Education program should be started at the hospital level along with conferences to spread knowledge about this disease.

## Introduction

Cervical cancer is one of the leading causes of morbidity and mortality amongst the gynecological cancers worldwide [1]. In today's world, cervical cancer is primarily a disease found in low-income countries [2]. Of the nearly 500,000 new cases that occur annually, 83% are in the developing world, as are 85% of the 274,000 deaths associated with cervical cancer [3]. The South Asian region harbors one fourth of the burden of cervical cancer [4]. In India alone there are an estimated 132,000 new cases and 74,000 deaths each year [4]. Most women with cervical cancer in these countries present with advanced disease, resulting in low cure rates [4]. Several factors contribute to high burden of disease and advanced stage at presentation including poor knowledge about the disease furthermore there is a lack of screening among general population.

The situation in Pakistan is largely unknown. With the scarcity of epidemiological data, the only information available is through institutional and regional cancer registries, which may not be representative of true burden [5], [6], [7]. Based on one such registry in urban setting cervical cancer was responsible for 3.6 percent of cancer mortality [8]. In another study, it was reported that only 5 percent of women in Pakistan were aware of screening and only 2.6 percent of women actually had pap smear once a life [9]. Moreover screening is not available in most parts of the country and routine pap- smear is not even done in gynecological practice.

Causal role of infection with high risk Human Papilloma Virus (HPV) strains in cervical cancer has been targeted in the past two decades. A number of Primary and Secondary preventive approaches have been developed to prevent and treat infection with HPV [10]. High income countries have successfully reduced the cervical cancer burden by over 70 percent using one such approach of organized cytological based pap smears. A number of preventive strategies are currently being practiced in developed countries including use of two novel prophylactic vaccines and a number of secondary preventive strategies. Most of these interventions are currently not feasible in low income countries because of the already limited health care infrastructure [11]. At the same time it is imperative that our health care professionals are aware of these advances and especially of those interventions which can be utilized in low-resource settings.

Despite the active role which health care professionals have to play in preventing and educating about cervical cancer, to our knowledge no such study has been conducted which explores the current awareness about cervical cancer. In this study we aim to access the current knowledge amongst interns and nursing staff about cervical cancer and its prevention. The findings from this study will be useful at the policy level to complement knowledge and awareness about this important public health issue.

## Materials and Methods

### Study Design

A cross-sectional, interview- based survey was conducted in three major teaching hospitals in Karachi, Pakistan. The hospitals were Aga Khan University Hospital (AKU), Liaquat National Hospital (LNH) and Jinnah Post Graduate Medical Centre (JPMC). AKU is a privately run institution, LNH is semi-private and JPMC is under government administration.

Interns and nurses working in the above-mentioned hospitals, who gave written consent, were interviewed. Doctors other than the interns, any female with the history of cervical cancer, participants who did not give written consent and those rotating in Obs/Gynea were excluded to avoid bias in the study.

The study was conducted in June, 2009.

### Sample Size

No significant regional study was found on the topic so sample size was calculated taking prevalence to be 50%. Calculated size of sample came out to be 384 rounded off to at least 400. As no definitive data was available concerning the number of registered interns and nurses at each center, sample of 400 was divided based on convenience sampling. Greater number of study population was available at AKUH as compared to LNH and JPMC, thus larger number of the respondent were from AKUH in comparison to the other two.

### Data collection tools and Analysis

Questionnaire was designed based on the study objectives, taking help from the previous literature and studies available on the topic added with content specific questions. The questionnaire was divided into 2 main parts, first dealing with the socio-demographic profile of the subjects (eg. age, sex, education, etc.) and second consisted of the questions regarding the knowledge and awareness about different aspects of cervical cancer. Open ended question was asked about the risk factors and presenting features of cervical cancer with multiple responses. Similar responses were grouped together for the ease of presentation and understanding as described in the results.

Data entry was done through EpiData. Twice, two members of the team entered the same data and the data files were compared to rule out errors in entering the data. Analysis was done using on SPSS version 11. Percentages and proportions were calculated for all the variables. Relevant tables and graphs were computed.

### Ethical Consideration

Ethical approval was taken from AKU ethical review committee. Respondents were ensured about the confidentiality, they were briefed that their participation is voluntary and they have full right to withdraw from the study at any point.

## Results

There were 400 interview-based questionnaires filled. One hundred and eighty-four health professionals were from AKU, 111 from LNH and 105 from JPMC. There were in total 123 males with the mean age of 26.8±4.6 and 277 females with the mean age of 25.6±4.5. Sample consisted of 257 nursing staff and 143 interns. Of all the nurses interviewed, 82% had completed General Nursing Diploma, 17% had acquired BScN degree whereas 1.6% had MScN degree. Most of the interns interviewed were practicing either in medicine (55.1%) or surgery (36.4%).

Of all the interviews conducted 1.8% did not know cervical cancer as a disease, so they were excluded from further questioning.

### Knowledge about the Epidemiology of Cervical Cancer

The results showed that 23.4% of the sample correctly recognized that cervical cancer is the most common malignancy in gynecological cancers, while 52.0% thought that it's moderately common and 23.3% thought that it is least common. Greater number of the interns (29.5%) was aware of the correct answer in comparison to the nurses (19.5%).

Only 26.5% were aware that it is the second most common gynecological cancer leading to death. 1.8% was of the opinion that it contributes least to the mortality in gynecological cancers.

Overall only about 6.7% of the sample got both of the questions correct and were thus aware of the basic epidemiology of the cervical cancer (Table 1).

**Table 1:** Knowledge of the respondents regarding the epidemiology and etiology of cervical cancer.

Questions	Responses	N (%)
**How common is cervical cancer among gynecological cancers (n = 393)**	Least	**100(25%)**
	Moderately	**201(51%)**
	Most	**92(24%)**
**Mortality rank of cervical cancer amongst gynecological cancers (n = 393)**	Leading Cause	**54(14%)**
	Second In Rank	**102(26%)**
	Third In Rank	**95(24%)**
	Don't Know	**129(33%)**
	Others	**13(3%)**
**Causes of cervical cancer (n = 393)**	Genetics	**152(39%)**
(Multiple Responses Acceptable)	Infection	**306(78%)**
	Environmental	**53(13%)**
	Don't Know	**25(6%)**
**Organism causing the infection (n = 306)**	Bacteria	**69(23%)**
(Multiple Responses Acceptable)	Virus	**189(62%)**
	Fungus	**47(15%)**
	Parasite	**3(1%)**
	Don't Know	**32(10%)**
**Virus responsible for cervical cancer (n = 189)**	HPV	**116(61%)**
	Don't Know	**74(39%)**

### Knowledge about the Etiology of Cervical Cancer

Seventy-eight percent were aware that infection is one of the causes of cervical cancer, 39% said genetics and 13% opted for environment. Only a small percentage (6%) of the sample said that they “don't know” the cause of cervical cancer and thus they were not asked any further question relating to the etiology. (Table 1) Of all the study population who opted for infection, 62% said virus is the cause of that infection. Others responses were Bacteria (23%), fungus (15%) and parasite (1%). Sixty-one percent of the respondents who opted for virus were aware that HPV is that virus. They were then asked further question to evaluate their knowledge about HPV (Table 2).

**Table 2:** Knowledge about HPV – A causative agent for Cervical Cancer.

Questions	Responses	N (%)
**HPV is transmitted by : (n = 116)**	Oro-fecal	**4(3%)**
**(Multiple Responses Acceptable)**	Sexual	**103(89%)**
	Blood	**12(10%)**
	Environmental	**5(4%)**
	Don't Know	**5(4%)**
**Do you know HPV can cause any other disease? (n = 116)**	Yes	**64(55%)**
	No	**52(45%)**
**What disease? (n = 64)**	Wart	**45(70%)**
	Others	**19(30%)**
**Do you think HPV can be detected? (n = 116)**	Yes	**106(91%)**
	No	**10(9%)**
**Technique available for HPV detection: (n = 106)**	Blood Test	**13(12%)**
**(Multiple Responses Acceptable)**	Pap Smear	**65(61%)**
	PCR	**44(41%)**
	Biopsy	**21(20%)**

Majority of the respondents, who said HPV, were aware that it is transmitted sexually (89%), while few thought that blood (10%), oro-fecal (3%) and environment (4%) are also possible modes of transmission. Nearly half (55%) of all who knew about HPV, were aware that HPV can cause some other disease apart from cervical cancer. Cervical Wart (70%) was the most common disease quoted; others were penile cancer, pox disease, lymphoma, and other cancers. Ninety-one percent knew that HPV can be detected but out of these only 41% were aware of the correct technique to detect it, which is PCR. Majority of the interns and nurses thought that Pap smear (61%) can be used to detect HPV (Table 2).

### Knowledge about the Risk Factors and Presenting Features of Cervical Cancer

More than 35 risk factors were reported. Sexual practice, which included unprotected sex, multiple partners and other promiscuous behavior was the most common risk factor observed (45%). Sixteen percent of the sample was of the opinion that poor hygiene can be a risk factor for cervical cancer. Other stated risk factors were, infection, nulliparity, multiparity, early stage at first coitus, family history, smoking, age, contraception, etc (Table 3). Most common presenting complain reported was lower abdominal pain (42%) and per vaginal bleeding (40%) (Table 4). While few thought discharge (20%), fever (15%) and menstrual irregularity could also be the initial symptoms patients with cervical cancer can present with.

**Table 3:** Percentage distribution of risk factors for cervical cancer as reported by the study population.

Risk Factors	Number (%)
**Sexual Practice**	125 (45%)
**Infection**	55 (20%)
**Poor Hygiene**	44 (16%)
**Early age at first coitus**	31 (11%)
**Family History**	24 (8%)
**Smoking**	21 (7%)
**Multiparity**	20 (7%)
**Old Age**	13 (5%)
**Contraception (IUCDs, OCPs)**	13 (5%)
**Genetics**	12 (4%)
**Nulliparity**	8 (3%)
**Don't Know**	11 (4%)

**Table 4:** Percentage distribution of presenting features based on the responses of study population.

Presenting Feature	Number (%)
**Lower abdominal pain**	129 (42%)
**Bleeding Per Vaginal**	122 (40%)
**Discharge Per Vaginal**	60 (20%)
**Fever**	45 (15%)
**Menstrual Problems**	39 (13%)
**Weight Loss**	19 (6%)
**Itching**	19 (6%)
**Swelling (of cervix)**	13 (4%)
**Postcoital Bleeding**	10 (3%)
**Weakness**	8 (2%)
**Anemia**	4 (1%)
**Don't know**	89

### Knowledge about the Treatment of Cervical Cancer

Awareness about the best treatment of cervical cancer was widespread amongst study population. Seventy-two percent answered the correct treatment option which was “to treat according to the stage of the disease”. Twelve percent were of the opinion that only surgery is required to cure cervical cancer, whereas 6% were in favor of chemotherapy and 5% for radiotherapy, only.

### Knowledge about the Prevention of Cervical Cancer

Prevention of cervical cancer can be divided into primary prevention, which includes vaccine and secondary prevention, which includes screening test.

Fifty-four percent of both interns and nurses were aware that there is a screening test for cervical cancer and out of these 75% knew the correct screening test, which is Pap smear. Biopsy (8%), ultrasound (3%), HVS (2%) and Radiological scans (10%) were few of the incorrect responses observed. Majority of the interns and nurses were aware of the correct time to start screening which is after first coitus (37%) but very few were aware that Pakistani guidelines recommends screening after 3 years (10%) and not yearly (52%).

In total only 37 out of 393 were aware of the vaccine against HPV. Nearly all of the interviewees wanted to learn more about the vaccine (91%). Majority of the respondents opted for mass media (63%) and health professionals (63%) as a source through which knowledge concerning cervical cancer can be dissipated. Thirty-three percent found pamphlets to be a good source to obtain information from, whilst few were of the opinion that special lectures, conferences and seminars can also be useful.

## Discussion

The majority of respondents in our survey were unable to recognize cervical cancer as a major public health problem. Only about a quarter of the health professionals interviewed were able to correctly identify cervical cancer as being the most common gynecological cancer, as also reported for India(14). A similar number of study population identified cervical cancer as an important killer amongst gynecological cancers in women. This was an unexpected finding that the health professionals are unaware of the fact that cervical cancer is the second leading cause of cancer-related mortality among women in developed countries and is still the leading cause of death due to gynecological cancers in the developing countries (15, 11). This was on the contrary to the results of a study done in Uganda and Thailand to assess knowledge, attitudes and practices on cervical cancer screening among the registered nurses and medical workers, which showed that majority of the respondents were aware of the burden imposed by cervical cancer on health system and had moderate level of knowledge regarding cervical cancer and HPV [12]. Studies done on knowledge about cervical cancer, HPV infection and its prevention in general population show inadequate information of the participants on the concerned topic [13], [14], [15], [16], [17]. On the basis of these findings it can be expected that considering the knowledge about this disease in health professionals, the knowledge in general population of our country will be even less.

Only small number of participants was aware that HPV infection can lead to cervical cancer. These results again show insufficient knowledge of HPV infection being the cause of cervical cancer in health professionals in our country, even though 98% of cervical cancer in our part of the world is due to HPV infection, as reported in a study done in India [11]. These results are similar to other studies done in rest of the world showing less than satisfactory knowledge about the cause of cervical cancer in the community as well as the health professionals [12], [14], [15], [16], [18], [19], [20], [21], [22].

The respondents who were correctly able to identify HPV infection as cause of cervical cancer were further assessed about their knowledge about HPV itself. Eighty- nine percent correctly reported sexual contact as the mode of transmission for HPV infection. Less than half of the interviewees were knowledgeable about the information that HPV is also responsible for skin warts. In the view of 90% of the participants HPV could be detected but less than half correctly reported PCR to be the test used for detection of HPV infection. This shows that half of the study subjects who are aware of HPV infection and its relation to cervical cancer have moderate knowledge about HPV infection itself. These results show better knowledge about HPV infection in health professionals as compared to general population as shown by various studies done worldwide, but this information is still inadequate [14], [15], [16], [17], [18], [19], [20], [21], [22].

Risk factors for cervical cancer include early onset of sexual activity, multiple sexual partners, a high-risk sexual partner (eg, promiscuous sexual activity, sexual exposure to a partner with human papillomavirus infection), history of sexually transmitted diseases (eg, Chlamydia trachomatis, herpes simplex virus), smoking, high parity, immunosuppression, low socioeconomic status, prolonged use of oral contraceptives, and previous history of vulvar or vaginal squamous dysplasia [21], [23], [24], [25], [26], [27], [28], [29].

In our study population most of the health professionals stated, unsafe sexual practices, infection, early onset of sexual practice, as common risk factors which is in accordance with our literature search, however poor hygiene, family history and nulliparity as risk factors were some of the misconceptions present. The most frequently occurring symptoms at presentation are postcoital bleeding [30], abnormal vaginal bleeding, vaginal discharge that may be watery, mucoid, or purulent and malodorous. Pelvic or lower back pain and bowel or urinary symptoms mostly suggest of advanced disease.

From our study we found out that only 3% of the health professionals know postcoital bleeding as a common presenting feature of cervical cancer, although it is one of the pathognomic symptoms of cervical neoplasia. Eighty-nine respondents did not know of any presenting symptom of the disease, and one hundred and fourteen did not know about any risk factor. This emphasizes on the need to increase the awareness about cervical cancer in health professionals (interns and nurses) who are involved in the primary care of general patient population and form an important source of guidance for them.

Unlike most other cancers, cervical cancer is readily preventable when effective programs are implemented to detect and treat its precursor lesions [31]. Underutilization of cervical cancer prevention services by women in the high-risk age group of 30–60 years can be attributed to health service factors (such as lack of awareness of health professionals about the screening test and its guidelines, poor availability, poor accessibility, and poor quality of care provided), women's lack of information, and cultural and behavioral barriers. Our study also evaluated the knowledge of interns and nurses about Pap smear. Only 40% of the respondents were aware that Pap smear is the screening test for cervical cancer. This shows inadequacy of even our health professionals' knowledge about the secondary prevention of cervical cancer. This result can explain why most of the cervical cancer in the region presents late in advanced stage [8]. Other studies done in high and low income countries showed higher prevalence of awareness about the screening test. In a recent study in Pakistan, Imam SZ et. al. reported that the knowledge of availability of screening for cervical cancer amongst general population was only 5% and of the sample only 2.6% had ever received a Pap test [9]. One of the reasons for this finding could the lack of knowledge of health professional themselves, who are responsible to educate general population. Appropriate time of commencement of screening is after marriage as most of the females in Pakistan have their first intercourse after that. Most of the interns and nurses were aware of it but still a large number were of the opinion that screening could be initiated as late as menopause. This again could be a contributing factor towards late detection of cervical cancer and high risk of mortality associated with it. The frequency of screening is less for Pakistan as compared to other countries. Pakistani guidelines recommend Pap smear to be done every 3 years. Most of the respondents were aware that screening should be carried out every year as per American Cancer Society guidelines; very few knew that the interval can be extended to 3 years.

Prevention of HPV infections is very essential in prevention of cervical cancer. The advent of HPV vaccine has been a major breakthrough. Many studies have been done worldwide recently on the knowledge and awareness, attitude, beliefs and practices about HPV vaccine. The studies reported better knowledge in developed countries like the USA, Belgium and Australia, but other countries like Thailand, Turkey and China had poor information about HPV and HPV vaccine [12], [14], [32], [33], [34], [35], [36]. This vaccine will soon be introduced in Pakistan (by 2010). As expected very few of the respondents were aware of the vaccine against HPV, as it is currently not available in this part of the world. A pleasing result was that most of the health professionals wanted to know more about the vaccine.

### Study Limitations

Due to non- availability of a formal data base of nursing staff and interns, this study was based on convenience sampling, thus the professionals interviewed may not reflect the awareness and knowledge of the entire center. A second limitation is that the exclusion of rural areas from the sampling frame means that study findings are not generalisable to providers in these areas. However, future studies on cervical cancer and HPV should undoubtedly focus on this population, considering the even poor quality of screening, treatment and counseling services in rural Pakistan.

### Conclusion

This study highlights inadequate knowledge about cervical cancer and its screening amongst health professions. Continuing Medical Education program should be started at the hospital level along with seminars which highlight the importance of screening in women. Nursing staff especially if properly aware of this disease can educate masses and hence increase the health seeking behavior in women in Pakistan. More emphasis should be placed on curriculum taught in undergraduate education. Only through proper education of health care workers burden of cervical cancer can be reduced.
